# Efficacy of Mycophenolate in Steroid-Dependent and Frequently Relapsing Adult Minimal Change Disease: A Retrospective Cohort Study

**DOI:** 10.7759/cureus.77314

**Published:** 2025-01-12

**Authors:** Surendra S Rathore, Kumari Nirja, Sunita Choudhary

**Affiliations:** 1 Nephrology, Dr. Sampurnanand Medical College, Jodhpur, IND; 2 Physiology, Dr. Sampurnanand Medical College, Jodhpur, IND

**Keywords:** frequent relapsing nephrotic syndrome, immunosuppression, minimal change disease, mycophenolate, nephrotic syndrome, steroid-dependent nephrotic syndrome

## Abstract

Background

Just like in the pediatric population, minimal change disease (MCD) in adults is characterized by a high relapse rate following steroid therapy. A significant proportion of these patients become either frequent relapsers or steroid-dependent (SD). These SD or frequently relapsing (FR) adult MCD cases require additional forms of immunosuppressive therapy to avoid steroid-related adverse effects.

Aim

The primary objective of this study was to evaluate the efficacy of mycophenolate (MF) in maintaining complete remission in adult MCD patients with steroid dependence or frequent relapses. The secondary objective was to assess the safety and long-term outcomes of MF therapy in this patient population.

Methods

This retrospective cohort study included all adult biopsy-proven FR/SD MCD cases who were prescribed MF therapy during period of January 2018 till May 2024. MF drug was prescribed for 12 months duration in MF-responsive patients. After extracting baseline demographic data, these patients were retrospectively followed up to assess induction of remission and maintenance of remission after discontinuation of MF therapy.

Results

In the present study, 24 adult FR/SD nephrotic syndrome cases were included for analysis. MF therapy was able to maintain steroid-free remission for the whole duration of therapy (12 months) in 20 (83.3%) patients. Four patients were non-responsive to MF therapy and relapsed once steroids were withdrawn despite patients being on full dose of MF. Among 20 MF-responsive patients, there was no relapse of nephrotic syndrome in 14 (70%) patients (sustained remission) till the last follow-up, with a mean duration of 18.78±8.07 months. In six (30%) cases, there was a relapse of nephrotic syndrome after MF drug was stopped, with a mean interval of 10.16±3.65 months.

Conclusions

Our study concludes that MF is effective in steroid-sparing in adult FR/SD MCD patients. The MF-based therapy was able to maintain complete remission in 83.3% cases for the whole duration of therapy. Moreover, in 70% of 20 MF responsive patients, the 12 months of MF therapy achieved sustained remission till the last follow-up.

## Introduction

Minimal change disease (MCD) accounts for 10-15% of nephrotic syndrome (NS) cases in adults [1]. Similar to nephrotic children, glucocorticoids till date remain the cornerstone of initial immunosuppressive therapy for adult patients with MCD as well. However, sustained clinical remission after initial steroid therapy can be attained in only less than 25% of cases [2]. The disease has a high relapse rate (50-75%), and a significant proportion of cases become either frequent relapsers (11%-29%) or SD (14%-30%). [3-5]. Nephrotic patients who are steroid-dependent (SD) or experience frequent relapses require additional forms of therapy to circumvent steroid-related adverse effects. Despite the advancement in immunosuppressive armamentarium during the last three decades, the management of frequently relapsing nephrotic syndrome (FRNS)/steroid-dependent nephrotic syndrome (SDNS) remains one of the “Gordian knots” for nephrology community, waiting to be untied. Limited evidence suggests that calcineurin inhibitors (cyclosporine, tacrolimus), cyclophosphamide, rituximab, or mycophenolate (MF) may be used to treat FR/SD MCD [6]. In recent years MF, which is devoid of significant side effects as compared to other immunosuppressive drugs, has been successfully used to induce and maintain remission in pediatric SDNS/FRNS cases [7-8], but there is very little evidence of its efficacy in adult frequently relapsing (FR)/SD MCD patients [9]. In this retrospective cohort study, we report our experience regarding the use of MF in 24 adult patients with SD/FR MCD.

## Materials and methods

Inclusion criteria

All adult (>18years age) biopsy-proven FR/SD MCD cases who were prescribed MF therapy during period of January 2018 till May 2024 were included.

Exclusion criteria

All pediatric MCD patients, patients who received MF for less than four weeks, patients with steroid-resistant MCD, pregnant females, those intending to conceive or of childbearing age, and those unwilling to practice effective contraception were excluded from the study.

Study design

It was a retrospective cohort study. The demographic, prior immunosuppression, and laboratory parameters of patients at the time of initiation of MF-mofetil (MMF) treatment were retrieved from the case records. The data of subsequent follow-up visits of these cases were also analyzed to assess study outcome parameters. This study was conducted in accordance with the guidelines set out in the Declaration of Helsinki and ICH-Good Clinical Practice. The study protocol was approved by the Institutional Review Board (IRB reference number: SNMC/IEC/IIP/2024/plan/05).

Definition

The following definitions were used for the study purpose [6]:

Nephrotic syndrome: nephrotic syndrome was defined as proteinuria > 3.0 g/24 hours, edema, and hypercholesterolemia.

Complete remission (CR) of nephrotic syndrome: reduction of proteinuria to <0.3 gram/day or protein-to-creatinine ratio (PCR) to <300 mg/g and stable serum creatinine and serum albumin >3.5 g/dL.

Partial remission: a reduction in proteinuria to 0.3-3.5 g/day or PCR 300-3,500 mg/g and a decrease of >50% from baseline.

Relapse of nephrotic syndrome: reappearance of proteinuria of more than 3.5 g/day or PCR > 3,500 mg/g after CR has been achieved.

SDNS: defined as relapse of nephrotic syndrome during the tapering phase of steroid treatment or within two weeks after discontinuation of therapy.

Frequent relapsers: designates those patients who present with two or more relapses within a period of six months (or four or more relapses per 12 months).

MF dosing regimen

All FR patients received standard oral steroid therapy of 1 mg/kg/day at the time of relapse for a minimum of one month. After the achievement of remission, dose of steroids was gradually tapered to a dose of 5 mg per week and then completely stopped. After attaining CR, MMF/MF-sodium was added to steroid therapy. The starting dose of MMF was 1,000 mg/12 h (or 720 mg/12 h of MF-sodium), which was tapered to 500 mg/12 h (360 mg/12 h of MF-sodium) after 6 months of CR. The MF therapy was continued for a minimum of 12 months after attaining CR. All cases were then serially followed up for any relapse of nephrotic syndrome.

In SDNS cases, the minimum effective dose of steroid was continued to maintain remission. The MMF drug was added to steroid therapy at a starting dose of 1,000 mg/12 h (or 720 mg/12 h of MF-sodium). The minimum effective dose of steroids was continued for the next four to six weeks and then gradually tapered off. After six months of MF therapy, the dose of MF was reduced to 500 mg/12 h (360 mg/12 h of MF-sodium) if CR was maintained. MF was continued for a minimum of 12 months after CR. All cases were then serially followed up for any relapse of nephrotic syndrome after stoppage of MF.

Statistical analysis

The categorical variables are expressed as numbers (percentages), while continuous data are reported as mean ± SD. Dichotomous variables were analyzed using Fisher’s exact test, and the unpaired t-test was used in case of continuous variables. A p-value of <0.05 was considered statistically significant. All calculations were performed using SPSS Version 26 (IBM Corp., Armonk, NY, USA).

## Results

Between January 2018 and May 2024, a total of 27 renal biopsy-proven adult MCD patients with FRNS/SDNS were prescribed MF therapy. However, follow-up records of three cases were not available; hence, the remaining 24 cases were included for analysis. The baseline clinical characteristics are reported in Table 1. Among these patients, the male/female ratio was 14/10, with a mean age of 42.75±17.11 (range: 21-70 years). This male predominance of MCD in our study is in accordance to the available literature [1,9]. Majority of patients belonged to the FRNS category (19 cases; 79.2%). Most cases had a history of nephrotic syndrome of prolonged duration prior to starting MF therapy (4.27±3.29 years; range: 8 months to 9 years) with multiple relapses (mean: 4.45±1.47 years). All but one patient had received one or other type of immunosuppressive therapy in addition to steroids.

**Table 1:** Baseline demographic and clinical features of study patients (N=24). CNI, calcineurin inhibitors; FRNS, frequently relapsing nephrotic syndrome; MF, mycophenolate; SDNS, steroid-dependent nephrotic syndrome

Variable	N (%) or mean±SD
Male	14 (58.3)
Female	10 (41.7)
FRNS	19 (79.2)
SDNS	5 (20.8)
Age of onset of nephrotic syndrome (years)	42.75±17.11
Mean duration of nephrotic syndrome prior to MF therapy (years)	4.27±3.29
No. of relapses prior to MF therapy	4.45±1.47
Cyclophosphamide use prior to MF therapy	14 (58.3)
CNI use prior to MF therapy	12 (50)
Rituximab use prior to MF therapy	4 (16.7)

The clinical response to MF therapy is enumerated in Table 2. MF therapy was able to maintain steroid-free remission for the whole duration of therapy (12 months) in 20 (83.3%) patients. Four patients (three SDNS cases and one FRNS case, with a mean age of 39.8±8.13 years) were non-responsive to MF therapy as evident by the fact that there was a relapse of nephrotic syndrome once steroids were withdrawn despite patients being on full dose of MF. All these four cases had earlier received cyclophosphamide and calcineurin inhibitors. In these cases, steroids were restarted and MF was stopped. None of the patients experienced any significant side effects in the present study. Five cases had mild gastrointestinal disturbances, but in none of the patients, these were severe enough to discontinue the drug.

**Table 2:** Clinical response to mycophenolate therapy (N=24). *Proportion is out of 20 patients who were responsive to MF therapy. CR, complete remission; FRNS, frequently relapsing nephrotic syndrome, steroid-dependent nephrotic syndrome; MF, mycophenolate; NS, nephrotic syndrome; SDNS, steroid-dependent nephrotic syndrome

Variable	N (%) or mean±SD
CR maintained while on MF	20 (83.3%)
Relapse while on MF therapy	4 (three cases of SDNS and one case of FRNS)
Sustained CR after stopping MF*	14 (70%)
Follow-up duration of sustained CR (in months)	18.78±8.07
Relapse of NS after stopping MF*	6 (30%)
Timing of relapse post-MF therapy (in months)	10.16±3.65

The follow-up records of the MF-responsive 20 patients were analyzed to assess whether MF therapy was able to maintain sustained CR or not. There was no relapse of nephrotic syndrome in 14 (70%) patients till the last follow-up (mean duration: 18.78±8.07 months [range: 8-40 months]). In six (30%) cases, there was relapse of nephrotic syndrome after MF drug was stopped. The mean interval of relapse post-discontinuation of MF was 10.16±3.65 months (range: 6-16 months).

Table 3 shows comparative analysis of the clinical characteristics of patients who were in sustained remission after discontinuation of MF (group 1) with the cases who relapsed after stopping the drug (group 2). There was no statistically significant difference in any clinical parameter between the two groups.

**Table 3:** Comparative analysis of characteristics between sustained remission (group 1) and relapsers after stopping MF therapy (group 2). Categorical variables were analyzed using Fisher’s exact test, while unpaired t-test was used in case of continuous variables. CNI, calcineurin inhibitor; FRNS, frequently relapsing nephrotic syndrome; MF, mycophenolate; SDNS, steroid-dependent nephrotic syndrome

Variable	Group1 (N= 14)	Group 2 (N=6)	p-Value
Age of onset of nephrotic syndrome	44±18.28	45.33±17.45	0.88
Sex (male)	8	4	1.00
FRNS	13	5	0.52
SDNS	1	1	0.52
No. of relapses prior to MF	4.42±1.39	5±1.67	0.43
Prior cyclophosphamide	5	5	0.14
Prior CNI	5	4	0.33
Prior rituximab	1	1	0.52

## Discussion

MCD, in contrast to the pediatric population where it is responsible for approximately 80% of total nephrotic cases, accounts for only 10% of nephrotic syndrome in adults [1]. Because of a relatively low prevalence of this glomerular disease in adults, there has been scarcity of adequately powered studies, and current data guiding treatment decisions in adult MCD patients (especially FR/SDNS cases) are limited and often extrapolated from pediatric studies [10]. In both pediatric population and adult cases, corticosteroids are the first-line treatment for MCD. Adult-onset MCD is responsive to glucocorticoids in almost 80% of patients [2]. Akin to the pediatric population, MCD in adults is a typically relapsing disease with an incidence rate of 50-70%, with a high proportion of patients becoming either frequent relapsers (11%-29%) or SD (14%-30%) [3-5]. In such cases, the repeated courses of high-dose glucocorticoids are associated with a wide range of serious side effects and are not desirable.

The clinical problem of SDNS/FRNS has been frustrating to the nephrology community till date due to concern of side effects of prolonged immunosuppressive agents. The main goal of treatment for these patients is to minimize the systemic toxicity of immune-suppressive therapies while avoiding further relapses. Traditionally, cyclophosphamide, cyclosporine, or tacrolimus is used as an alternative agent in steroid-sparing therapy [2,6,11-16]]. Although these drugs are effective in steroid-sparing, sustained remission is rarely obtained, and most of the patients relapse shortly after discontinuation of drug [14,16]. Furthermore, long-term administration of these medicines itself has important undesirable side effects. Thus, further studies are needed to improve the efficacy/toxicity balance of current available immunosuppressive agents in FR/SD-NS.

Among all currently available second-line immunosuppressive agents, MF seems to be the most suitable steroid-sparing agent since it has the least long-term systemic toxicity; hence, it may be used for longer durations without unfavorable side effects. In recent years, MF has been reported to be capable of inducing sustained remission of SDNS/FRNS in children [7, 8]. Unfortunately, there is a scarcity of literature on the use of MF in adults FR/SD-NS MCD patients [9]. In earliest documented use of MF in adult MCD patients, a complete steroid withdrawal was possible in five out of six SD patients [17]. In another case series, MF-based treatment was successful in the discontinuation of steroid treatment in four out of five cases of adult SD/FR MCD patients [18]. Seemingly, MF therapy plus half standard-dose prednisolone therapy was shown to induce remission with reduced rates of HBV reactivation in a study conducted on hepatitis B carriers [19]. In a retrospective study by Sandoval et al., the combination of MF and reduced dosage of prenisolone regimen was able to achieve remission in 27 (93.1%) out of 29 cases [9]. Moreover, MF plus prednisolone therapy led to sustained remission in 55% patients after a mean follow-up of 32.8 months. Interestingly, all patients who relapsed following MF discontinuation were found to be responsive to the second course of the same regimen.

Nonetheless, good-quality evidence on the use of MF in adult MCD remains elusive. We hope our study will help fill the gaps in our knowledge regarding the use of MF in adult FR/SD MCD cases. In the present study, the MF drug was shown to maintain CR for the whole duration of drug therapy in 20/24 adult MCD cases, while in four cases, there was a relapse of nephrotic syndrome once steroids were completely withdrawn. The results seem to be positive since MF as a sole immunosuppressive agent was effective in 83% adult MCD patients. Among the 20 MF-responsive cases, sustained remission was observed in 14 (70%) patients till the last follow-up. Most of the patients had a fairly long follow-up period in our study (mean duration: 18.78±8.07 months [range: 8-40 months]). Hence in 70% of MF-responsive patients, 12 months of MF course was able to induce sustained remission, which is the ultimate goal of treatment in nephrotic syndrome. In six (30%) cases, there was a relapse of nephrotic syndrome after MF drug was stopped. We performed comparative analysis of the clinical characteristics of patients who were in sustained remission after discontinuation of MF with the relapsing patients. There was no statistically significant difference in any clinical parameter between the two groups. Therefore, in the present study, we could not ascertain any clinical parameter that could predict the risk of further relapse after successful MF therapy.

The present study was not devoid of certain limitations. Being retrospective in nature, the results of this study cannot be generalized, and further prospective cohort studies are required to truly ascertain the role of MF therapy in adult MCD patients. The reporting bias of adverse effects documentation cannot be eliminated due to the retrospective nature of the study. Similarly, selection bias evaluation was also not possible in the present study. For example, it was not possible to ascertain during the study period how many adult FR/SDNS MCD patients who were eligible for MF therapy as per the criteria laid down in the Methods section were not provided MF therapy due to various reasons.

## Conclusions

The present retrospective cohort study concludes that MF is a safe and effective steroid-sparing second-line agent in the treatment of adult FR/SD MCD patients. After attaining CR with oral steroids, the MF-based therapy was able to maintain remission in majority of cases for the whole duration of therapy. Hence, a one-year course of MF drug was effective in obviating further doses of steroids and helped curtail the cumulative dose of steroids. Moreover, in most MF-responsive patients, the one-year course of MF therapy achieved sustained remission till the last follow-up of these patients, a parameter that is the ultimate goal of treatment in nephrotic syndrome cases.
